# Lectures on the Principles and Practice of Medicine

**Published:** 1852-08

**Authors:** 


*Lectures on the Principles and Practice of Surgery.* By BRANSBY B. COOPER, F. R. S., Senior Surgeon to Guy's Hospital, etc. Philadelphia: Blanchard & Lea. 1852. 8vo. pp. 771.

This volume embraces a series of lectures delivered before the pupils of Guy's Hospital, London. It does not profess to be a systematic work on the Elements of the Science of Surgery, but a compendium "in which the student may meet with a clear account of the practice of that science, established, not only on the author's own experience, but likewise upon the best acknowledged authorities." It is evidently a practical work, for which the large experience of the author, having been surgeon to Guy's Hospital for twenty-five years, must furnish abundant materials. As such it will commend itself to the surgical practitioner.

